# Complete Laparoscopic Ovarian Cystectomy of Giant Ovarian Serous Cystadenoma

**DOI:** 10.7759/cureus.33901

**Published:** 2023-01-17

**Authors:** Indira Prasad, Sudwita Sinha, Upasna Sinha, Mukta Agarwal

**Affiliations:** 1 Obstetrics and Gynaecology, All India Institute of Medical Sciences, Patna, Patna, IND; 2 Radiology, All India Institute of Medical Sciences, Patna, Patna, IND

**Keywords:** laparoscopic technique, serous cystadenoma, giant ovarian tumors, laparoscopic cystectomy, minimally invasive surgery

## Abstract

We report here a case of an unmarried teenage girl with a 19-kg giant ovarian cystic tumors of size 37cm×31cm×22cm, which was presumably benign on imaging and with negative tumor markers; treated by complete laparoscopic ovarian cystectomy following decompression and the patient was discharged the next day.

## Introduction

It is common to encounter cystic abdominopelvic tumors in clinical practice, but it is very rare to find a giant ovarian cyst in today’s world [1]. With the advent of new imaging modalities with excellent resolution and sensitivity, ovarian cysts are detected at very early stages and much smaller sizes [2]. But occasionally, they do reach enormous dimensions without many symptoms. In literature, a few such cases of giant ovarian cysts have been sporadically reported [1-5]. The patient’s age, size of the cyst, and histopathological nature are the determining factors in deciding the management of ovarian masses [1-5]. Conservative surgeries such as ovarian cystectomy and oophorectomy are adequate for benign lesions [3]. Open laparotomy or combined laparoscopy and laparotomy approaches are still the most followed surgical methods used for huge ovarian cysts. However, complete laparoscopic surgery is more suitable for benign cysts management with better cosmetic outcomes and shorter hospital stays.

## Case presentation

A case of an unmarried 19-year-old teenage (nulliparous) girl with giant ovarian serous cystadenoma weighing 19 kg is reported here who presented to Gynecology OPD with gross distension of the abdomen for the past one year. Increasing abdominal girth was accompanied by vague abdominal pain and respiratory discomfort which worsened on lying down. There was accompanying insomnia, anorexia, and weight loss. There was no history of vomiting or other gastrointestinal symptoms, urinary symptoms, colicky pain, or fainting attacks. She had no previous history of illnesses, allergies, or surgery. She denied the use of any medications. There was no family history of malignancies. She had no previous investigations and was from a poor family. Her menarche commenced at the age of 11 years with subsequent regular cycles. Her body weight was 49.5 kg, her height was 151 cm, and her BMI was 21.71 kg/m^2^. On physical examination, she was afebrile with a pulse rate of 84 beats/min, blood pressure of 120/72 mm Hg, and respiratory rate of 24 breaths/min. There was no jaundice, edema, or lymphadenopathy. Secondary sexual characteristics were evident. Abdominal examination revealed a grossly distended abdomen of size bigger than a 36-week gravid uterus that appeared more like ascites with demonstrable fluid thrill as no definite lump could be palpated through the tense abdomen whose girth was 112cm. On percussion, a dull note was appreciated, and no tenderness was noted. Bowel sounds were normal. The external genital examination was normal.

Pregnancy could have been one possibility in a young girl with a distended abdomen which was ruled out as there was no history of amenorrhea. Organomegaly was excluded from clinical examination. Uterine mass was not a possibility as there was no history of menstrual irregularities. So ovarian cyst was the most likely diagnosis.

**Figure 1:**
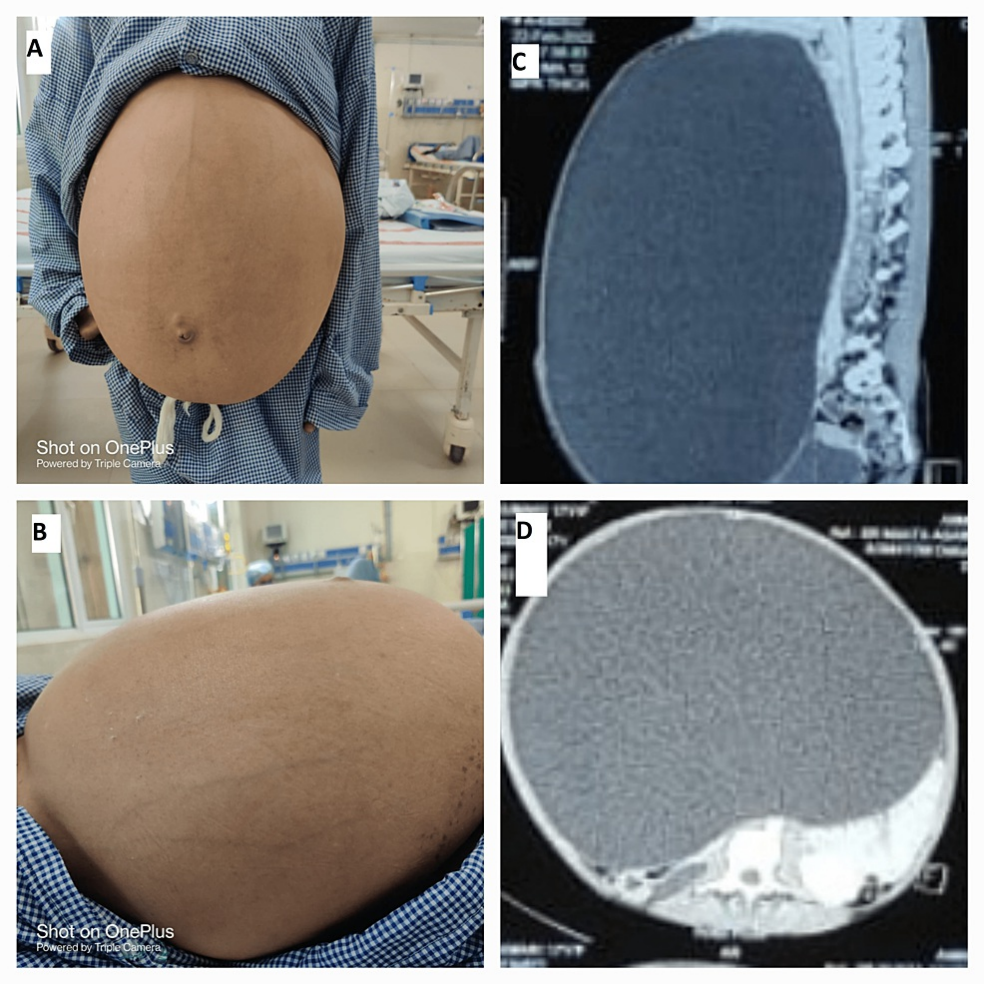
(A) Front view of patient’s abdomen before surgery. (B) Lateral view of patient’s abdomen before surgery. (C) CT imaging showing sagittal sections of giant ovarian cyst. (D) CT imaging showing axial sections of giant ovarian cyst.

A plain radiograph of the chest was within normal limits. Transabdominal ultrasonography revealed a large abdominopelvic cystic mass extending from the pubis to the epigastrium with a mass effect on abdominal organs and with no evidence of solid components or septations. The uterus was normal and endometrial thickness was 10 mm. Serum CA-125 was 5.4 IU/mL, Serum CEA was 1.5 IU/mL, Serum CA 19-9 was 10.67 IU/mL, Serum Alpha-fetoprotein was 1.3 IU/mL and total HCG was 2.0 IU/mL; all tumor markers were within normal range except serum LDH, which was 685 U/L. Abdominopelvic computerized tomography (CT) findings were consistent with a large well-defined homogeneously cystic lesion 37cm×31cm×22cm (in craniocaudal, transverse, and anteroposterior dimensions) in the abdomen arising from left ovary while right ovary, adnexa, and uterus were unremarkable. Rest all other organs were unremarkable except that left hydroureteronephrosis was noted most probably due to the mass effect of the tumor. As no solid component was noted in the lesion, no abdominopelvic metastases or lymphadenopathy was noted in either ultrasound or CT scan, the possibility of the tumor being malignant was negligible. Considering the cyst to be benign in this unmarried girl, we decided to manage it with laparoscopic cystectomy. Figure 1A shows a front view of the patient's abdomen before surgery; Figure 1B shows a lateral view of the patient's abdomen before surgery; Figure 1C shows sagittal sections of CT imaging; and Figure 1D shows axial sections of CT imaging of giant ovarian cyst.

**Figure 2:**
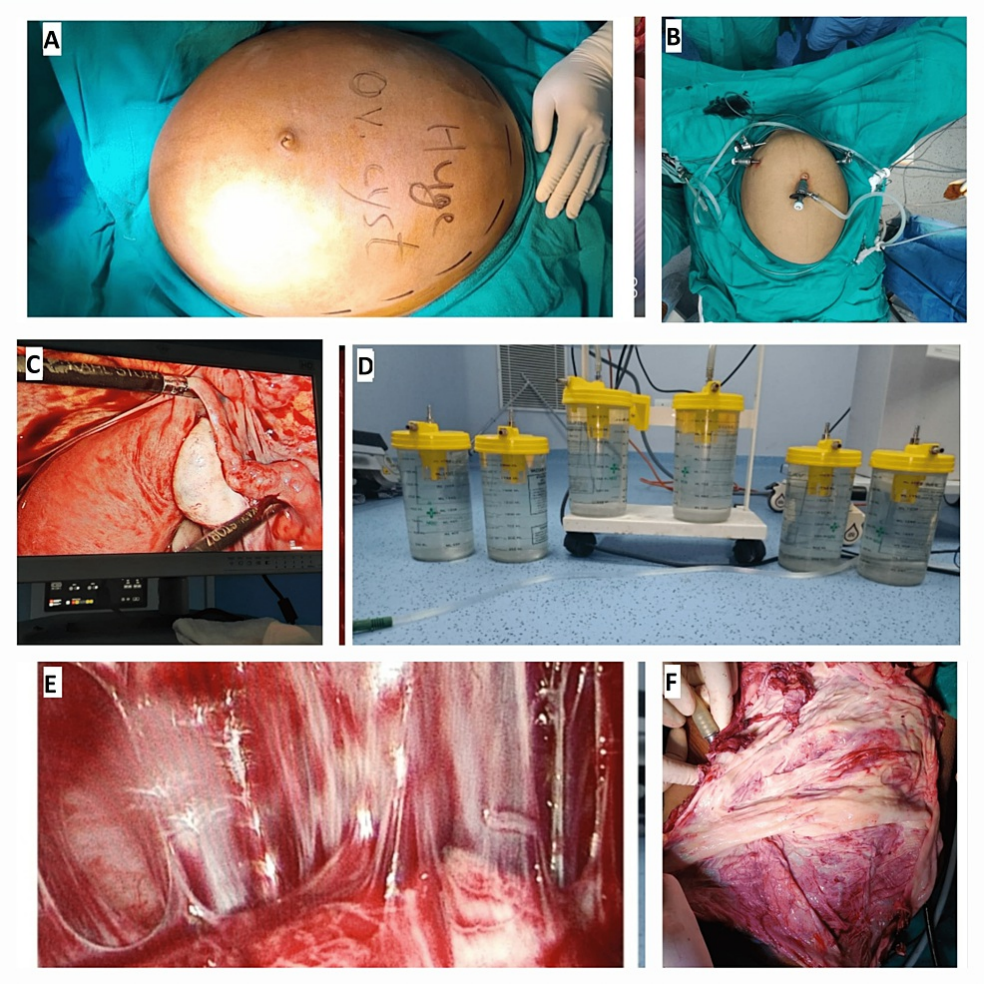
(A) View of patient’s abdomen before insertion of laparoscopic trocars. (B) View of patient’s abdomen after insertion of laparoscopic trocars. (C) Laparoscopic view showing normal uterus, right fallopian tube and right ovary. (D) View of jars containing clear watery fluid drained from cyst. (E) Laparoscopic view of cystectomy being done. (F) View of cyst wall after specimen retrieval.

After adequate counselling and informed consent, the patient was taken up for laparoscopic cystectomy. Open-entry laparoscopy was performed with an approximately 1 cm incision on the upper border of umbilicus, in which a 10 mm trocar was placed directly into the cyst and a suction catheter was attached to the trocar cannula following which around 16 litres of clear watery cyst fluid was drained. After this the umbilical cannula was repositioned and a stay suture was taken on the cyst wall margins. The telescope was placed via umbilical 10 mm port. A pneumoperitoneum pressure of 12-14 mmHg was achieved and was maintained throughout the procedure. Three ancillary 5-mm trocars were then placed under vision, two on the left side and one on the right side of the umbilical port. The surgery was performed under general anesthesia with the patient in the lithotomy position. Throughout the procedure, the surgical table was put in the Trendelenburg position, but the angle was modified in different phases of the surgery as per the anesthesiologist’s needs. Huge cyst after decompression was still quite big and was seen arising from the left ovary. Right ovary, right fallopian tube and uterus were found to be absolutely unremarkable. Through the laparoscope, the abdominal cavity could only be partially visualized because the giant cyst was in the way and the cyst’s origin was traced with difficulty and it was found to be arising from the left ovary. Following this, cystectomy was performed using bipolar forceps and scissors while conserving the ovarian tissue on the left side. The huge cyst wall was extracted completely through the 10-mm umbilical port, and it weighed about 1,150 g. A 5-mm telescope was used which was introduced through one of the side ports. A 10-mm grasper was then introduced through the 10-mm umbilical port and cyst wall was grasped under vision and brought outside the umbilical skin incision for extraction. The final volume of the drained cyst fluid content was 15,950 mL. Afterward, pneumoperitoneum was induced again, and systematic visual inspection of the abdominal cavity was done. The liver, gallbladder, spleen, and diaphragm appeared normal, and no macroscopic signs of malignancy were noted. No blood loss or other intraoperative complications occurred. The total operative time was 150 min. Patient tolerated the procedure well and had an uneventful recovery. Figure 2A depicts laparoscopic view showing normal uterus, right fallopian tube and right ovary; Figure 2B shows image of patient's abdomen before insertion of laparoscopic trocars; Figure 2C shows image of patient's abdomen after insertion of laparoscopic trocars; Figure 2D shows cyst wall after specimen retrieval; Figure 2E shows laparoscopy view of cystectomy being done and Figure 2F shows image of jars containing clear watery fluid drained from cyst.

**Figure 3:**
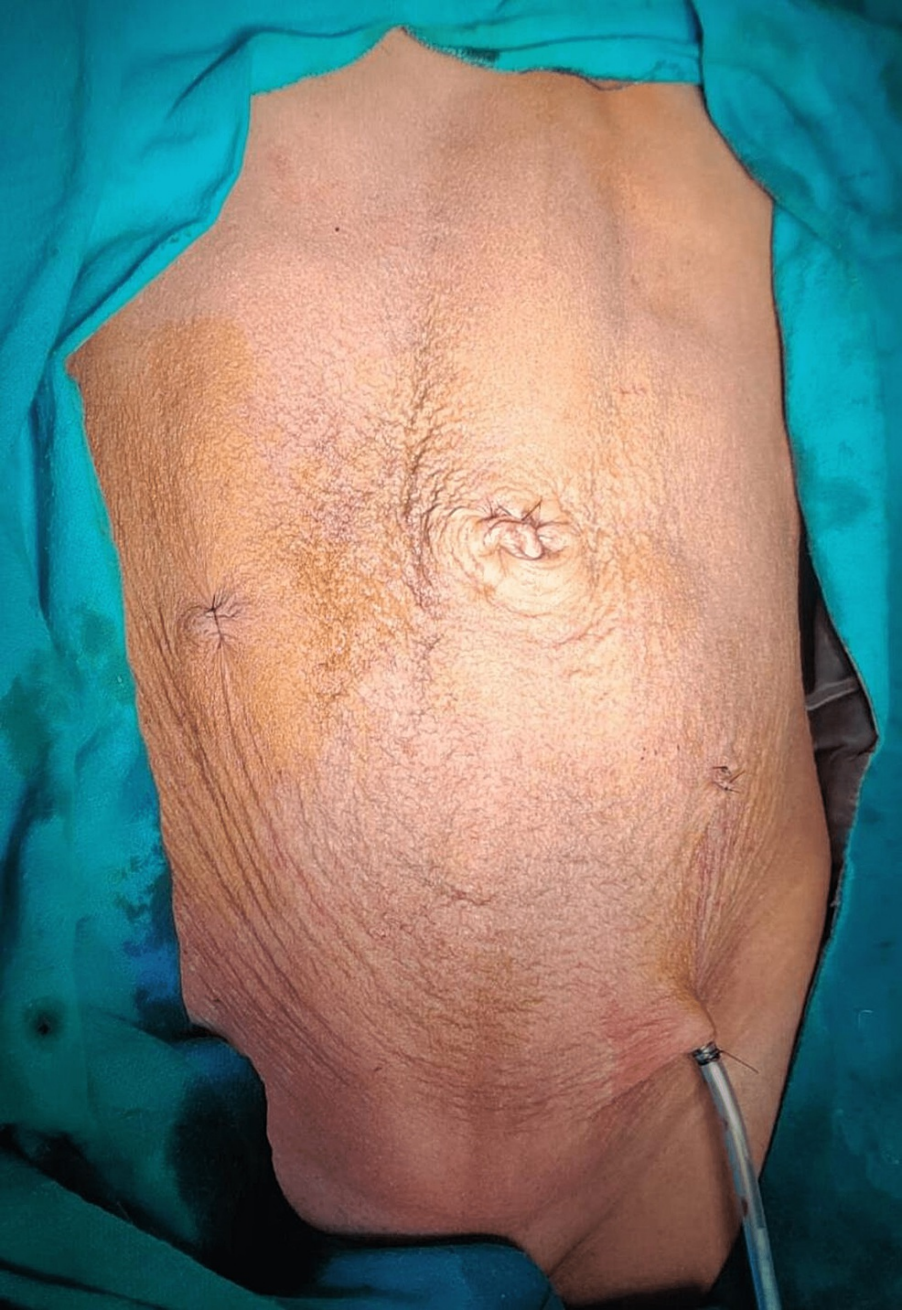
View of patient’s abdomen after surgery.

The patient started to ambulate on the same day of surgery, passed urine after self-retaining catheter was removed in 12hrs and was discharged the next day. No malignant cells were reported on cytology examination of the cyst fluid and the cyst wall was reported as serous cystadenoma on histopathology (Figure 3).

## Discussion

Ovarian tumors involve a variety of histological tissues, of which epithelial tissues are the most common. Eighty percent of all epithelial tumors are benign. Serous cystadenomas are the most common cystic ovarian tumors. In the above case, probably due to non-troubling symptoms, the patient did not seek medical attention early which led the tumor to enlarge and reach a giant size. Socioeconomic factors also contributed to her late medical consultation. The absence of solid nodules, ascites, and increased vascularity on imaging and negative tumor markers pointed toward a diagnosis of a benign tumor, which helped in deciding the choice of surgery for this patient. Laparoscopic cystectomy with no spillage and preservation of ovarian tissue is the management of choice in a benign ovarian cyst where fertility is desired, and this is what was done in the above case. There is no clear consensus or proper guidelines regarding the upper limit of the size of the tumor that can be operated via minimally invasive surgery as there is a risk of capsular rupture, peritoneal spillage, and seeding in case the tumor turns out to be malignant or borderline. However, the advantages of laparoscopic surgery such as lesser postoperative pain, shorter hospital stay, better cosmetic outcomes, and early return to activities in addition to the advancing techniques of specimen retrieval in minimally invasive surgery to tackle peritoneal seeding have led to its increased preference over open surgery in benign cases.

To date, few such cases have been reported in the literature. Dolan et al. reported a giant ovarian cyst of 40.5cm×30cm×22.5cm which was decompressed by minilaparotomy followed by laparoscopic oophorectomy [3]. Sanjay et al. reported a case of a giant ovarian cyst measuring 58cm×46cm, weighing 27 kg in an 85-year-old lady, which was removed by infraumbilical midline incision after decompression of the cyst [4]. Baradwan et al. reported a giant ovarian cyst of 31.7cm×26.3cm×15.6cm weighing 11 kg, which was removed laparoscopically after decompression [1]. Sanna et al. reported laparoscopic left salpingo-oophorectomy of a giant ovarian tumor of size 24cm×15cm×10cm followed by removal by minilaparotomy [5]. Wong et al. also reported a case of complete laparoscopic removal of a giant ovarian cyst of size 21.4cm×17.7cm×7.1cm [2].

To our knowledge, this is the first case reported of complete laparoscopic management of a giant ovarian tumor more than 35 cm in the largest dimension where minilaparotomy was not done to remove the tumor.

## Conclusions

To conclude, complete laparoscopic surgery with decompression can be done for giant ovarian cyst removal provided the tumor seems benign on comprehensive workup. However, care should be taken to avoid spillage during decompression. Considering the advantages of minimally invasive surgery, the size of the tumor should not be a limiting factor in attempting minimally invasive surgery in seemingly benign ovarian cystic tumors. Various different ways and techniques for specimen retrieval have been described as not necessitating minilaparotomy. However, one should not also be overzealous in attempting laparoscopic surgery in a case where malignancy is not completely ruled out.
